# Drug Discovery Models and Toxicity Testing Using Embryonic and Induced Pluripotent Stem-Cell-Derived Cardiac and Neuronal Cells

**DOI:** 10.1155/2012/379569

**Published:** 2012-05-08

**Authors:** Rahul S. Deshmukh, Krisztián A Kovács, András Dinnyés

**Affiliations:** ^1^BioTalentum Ltd., 2100 Gödöllö, Hungary; ^2^Molecular Animal Biotechnology Laboratory, Szent Istvan University, 2100 Gödöllö, Hungary; ^3^Department of Farm Animal Health, Faculty of Veterinary Medicine, Utrecht University 3584 CL Utrecht, The Netherlands

## Abstract

Development of induced pluripotent stem cells (iPSCs) using forced expression of specific sets of transcription factors has changed the field of stem cell research extensively. Two important limitations for research application of embryonic stem cells (ESCs), namely, ethical and immunological issues, can be circumvented using iPSCs. Since the development of first iPSCs, tremendous effort has been directed to the development of methods to increase the efficiency of the process and to reduce the extent of genomic modifications associated with the reprogramming procedure. The established lineage-specific differentiation protocols developed for ESCs are being applied to iPSCs, as they have great potential in regenerative medicine for cell therapy, disease modeling either for drug development or for fundamental science, and, last but not least, toxicity testing. This paper reviews efforts aimed at practical development of iPSC differentiation to neural/cardiac lineages and further the use of these iPSCs-derived cells for drug development and toxicity testing.

## 1. Introduction

The history of induced pluripotency/cellular reprogramming dates back to the 1950s when Briggs and King developed a technique of nuclear transfer (NT) to test the developmental potential of late-stage blastula cells following transfer into enucleated *Xenopus* oocytes [1]. Later experiments [2, 3] with the transfer of nuclei of differentiated cells into the amphibian oocyte lead to the production of live offspring suggesting genetic/epigenetic reprogramming of the differentiated cells to the totipotent/pluripotent state. In mammalians, the first adult-cell-derived animal, Dolly the sheep, was produced using SCNT only in 1997 [4], strengthening the concept of epigenetic reprogramming of the differentiated mammalian cells to totipotent/pluripotent state mediated by the ooplasm. Several reports later proved that the oocyte contains certain factors which are responsible for the reprogramming of the transferred genome [5, 6].

 Moreover the fusion of the somatic cells with pluripotent stem cells such as embryonal carcinoma cells (ECCs) derived from teratocarcinoma [7, 8] was able to induce pluripotency in the somatic cells [9]. This suggests that the pluripotent cells contain certain factors which are responsible for the conversion of the somatic cells into the pluripotent state. Embryonic stem cells (ESCs) derived from the inner cell mass of mouse [10] and subsequently from human blastocysts [11] are considered pluripotent. Mouse ESCs are able to contribute to all kinds of tissues of the live offspring after injection into the blastocysts. The pluripotency of human and mouse ESCs have been shown in vivo by teratoma formation and in vitro by embryoid body differentiation assays to form tissue of the three germ layers (endoderm, ectoderm, and mesoderm) [12]. The expression of key transcription factors such as OCT4, SOX2, and NANOG is mandatory to keep these cells self-renewing and pluripotent. Fusion of ESCs with somatic cells has led to the conversion of somatic cells from differentiated to pluripotent state.

The conclusion from the above observations is that the pluripotent cells have certain regulatory pathways involving powerful transcription factors which are sufficient to revert the somatic cells to a pluripotent state. Screening of 24 different transcription factors by the Yamanaka group [13] surprisingly demonstrated that generation of induced pluripotent stem cells (iPSCs) required a combination of only four transcription factors: OCT4, SOX2, KLF4, and c-Myc (OSKM) [13]. These iPSCs were almost identical to ESCs at the molecular as well as morphological level. Generation of iPSCs has fundamentally changed stem cell research considerably. In the case of iPSCs derived from somatic cells, the ethical issues and immunocompatibility problems arising from use of ESCs for cell therapy can be avoided. Mouse and human iPSCs can therefore be used to study the early developmental process, disease mechanisms, cell therapy, drug discovery, and toxicity testing assays. Methods developed to produce the iPSCs with no/less genetic modifications are under examination for robustness and ease of use.

## 2. Methods to Produce iPSCs

Since the description of the first iPSCs from mouse [13] and human [14], several optimized methods have been developed to produce iPSCs from various tissues and species [15–20]. The c-Myc from the original OSKM factors was thought being responsible for tumorigenicity [21] and thus affecting the potential clinical use of the cells. Consequently, efforts were made to screen more transcription factors, and the iPSCs were produced using a set of transcription factors OCT4, SOX2, NANOG, and LIN28 suggesting alternative factors affecting the pathways needed for reprogramming [22–24]. iPSCs can also be generated with fewer transcription factors [25]. Initially the technology involved the use of retroviral or lentiviral vectors for the transduction of the reprogramming factors. This technique leads to the integration of the viral vectors in the genome causing insertional mutations, and these cells are not likely to be acceptable for the clinical purposes. This problem was partially overcome by the use of a single polycistronic lentiviral vector carrying all OSKM factors reducing the number of insertions in the genomes [26, 27]. Furthermore, the use of nonintegrative viral vectors has also been suggested [28].

Options towards delivering the reprogramming factors with less or no genetic modification include the use of LoxP sites and Cre-induced excision of the transgenes—this has been achieved successfully—while in case of transposon-mediated gene transfer the suggested excision by transposase of the integrated vector sequences has never been published [29–31].

Protein transduction can completely replace the need for gene delivery for the generation of iPSCs. The conjugation of proteins with the short peptides responsible for cell penetration can be used for delivery of the proteins into the cells. Mouse and human iPSCs were generated with this approach using purified polyarginine-tagged OCT4, SOX2, KLF4, and c-MYC proteins [32–34]. Yamanaka factors have also been introduced by an mRNA-transfection method, Although this approach is reported to be more efficient and is not based on integration into the host genome, there is still a very low but not negligible risk of genetic alteration given that the exogenous substance introduced into the cells is nucleic acid [35].

Though until recently the protein iPSCs was considered as the best approach, the use of microRNAs (miRNAs) with or without transcription factors has been shown to be an efficient method to generate these cells [36, 37]. The generation of iPSCs using only miRNAs without any transcription factors has created an interesting puzzle concerning the mechanisms of reprogramming [37]. Notwithstanding the enigmatic mechanism behind the miRNA-mediated reprogramming, presently this method offers the highest published yield of reprogrammed cells and does not modify the genome of the cells—qualifying miRNA-reprogrammed cells for potential clinical applications. Nevertheless, further evaluation and confirmations by independent teams will be needed to demonstrate the superiority of any given method—although all of them become commercially available (and patent protected) within months of their first publication, often without independent validation.

## 3. ESCs and iPSCs in Drug Discovery and Toxicity Testing

Developing reliable systems to study drug toxicity is a major challenge for developing new and safe drugs for the treatment of humans. Currently, toxicological testing is based on the established immortal cancer cells lines containing chromosomal abnormalities, primary explanted somatic cells, and laboratory animals. Immortalized cell lines, showing several features reminiscent of cancer, mimic neither the normal physiological status nor the diseased state of the organism in vivo. The heterogeneity of primary explant cultures leads to inconsistent results and low reproducibility in toxicity testing. Using live animal models for toxicity testing may not mimic the human physiology, can raise ethical/animal welfare concerns, and is rather expensive. Research on ESCs and iPSCs promises to enhance drug discovery and development by providing simple, reproducible, and cost-effective tools for toxicity testing of drugs under development and, on the other hand, for studying the disease mechanisms and pathways [38–40]. Modeling human disease in standardised cell culture and the opportunity for high throughput drug screening are potential advantages of using iPSCs [38]. Patient-specific iPSCs could improve the efficiency of drug discovery by helping the identification of drugs effective in specific patient populations.

The ESCs, and to some extent iPSCs, differentiated either into cardiac or into neural cell types have been used widely for drug discovery and toxicity testing. This application is the most advanced and practical use of pluripotent cells; however, the acceptance of major pharmaceutical companies to adopt new approaches and replace the well-established and FDA-approved test methods is a rather slow process [40–43]. Consequently, disease models developed from iPSCs have not been used for the development of drugs that have reached clinical trials. Some experiments and ongoing efforts will be the focus of the remainder of this paper.

## 4. ESC- and iPSC-Derived Neurons in Drug Discovery

Neurodegenerative diseases including Parkinson's disease (PD), Alzheimer's disease (AD), Huntington's disease (HD), and amyotrophic lateral sclerosis (ALS) constitute an increasing burden for society. The mechanisms of neurological disorders like AD and PD is not well known due to the limited accessibility of the diseased tissue. Several iPSC lines were derived from spinal muscular atrophy (SMA) [44], schizophrenia [45], familial dysautonomia (FD) [46], and Friedreichs ataxia (FA) patients [47] to study the disease progression. The differentiation potential of ESCs and iPSCs into functional neurons and glia is known, and the specific culture conditions needed for the differentiation of ESCs and iPSCs into neural lineage for optimization of drug discovery models are more or less established even though need further refinement.

The establishment of in vitro models of ALS by differentiation of ESCs into motor neurons was one of the pioneering examples [48–50] of neurological disease modeling. Transgenic mice carrying human superoxide dismutase mutation (G93A) responsible for ALS were used for ESC isolation. The major observation made by this study was the more rapid death of G93A-positive motor neurons than their wild-type counterparts. Moreover, the astrocytes in the G93A-positive cultures were involved in the secretion of toxic factors causing selective and increased death of motor neurons without having an effect on interneurons. Human ESC-derived motor neurons were also killed by the mutant mouse astrocyte-conditioned medium [49, 51]. This work has demonstrated the potential for successful disease modeling using ESC-derived neuronal and astroglial cells.

A group of neurodevelopmental defect related autism spectrum disorders (ASD) have also been studied using iPSCs [52], including the Rett syndrome caused by the X-chromosome linked MeCP-2 mutation. This mutation causes impaired neural development after one year of age and the affected individual shows symptoms similar to other ASDs. iPSCs generated from the affected individuals were differentiated into GABAergic inhibitory and glutamatergic excitatory neurons. Interestingly, erased X-inactivation of the MeCP-2 during reprogramming was reestablished in the differentiated neurons, an observation that highlights the great potential that exists in disease modeling by pluripotent cells, and also the need of producing the congruent cell-type by differentiation from pluripotent cells for the purpose of disease modeling. After 2 months—in culture no significant effect on survival of the MeCP-2 neurons was observed. However, the number of glutamatergic synapses decreased significantly as observed in the Rett syndrome. Interestingly, treatment of these neuronal cultures with the insulin-like growth factor 1 (IGF-1) has shown an increase in the number of synapses, pinpointing the fact that rescue experiments can successfully be designed based on cultures of neurons harboring a disease-specific mutation. Overall, this demonstrates the ability of patient specific iPSCs in helping understand complex neurodegenerative diseases and to serve as a model for drug discovery.

Another major neurological disorder PD, that affects a subset of midbrain dopaminergic neurons, is a sporadic and late-onset disease. Out of several identified mutations the most common PD associated mutation is in Leucine-rich repeat kinase-2 (LRKK2). iPSCs generated from patients carrying a LRKK2 mutation were differentiated down the neuronal lineage and physiologically active dopaminergic neurons were produced [53]. The comparative analysis of gene expression profile by microarray has shown that neurons produced from the patient iPSCs had higher expression of oxidative stress genes, compared to the control patient iPSCs. Moreover, the protein *α*-synuclein, responsible for formation of characteristic aggregates in PD was highly expressed [54]. These neurons were found to be susceptible to hydrogen peroxide and 6-hydroxydopamine like stressors. This experiment demonstrated that the patient specific iPSC-derived neurons can be used for neurological disease modeling.

Recently, FD, a genetic disease, caused by mutation in I*κ*B kinase complex-associated protein (IKBKAP) was studied by Lee et al., [46]. The disease leads to the death of neural crest derived neurons in sensory and autonomic ganglia. iPSCs were generated from the affected individual and were subjected to neuronal differentiation. The neural crest precursors derived from these iPSCs showed low level of IKBKAP protein as well as defective migration and neuronal differentiation. The treatment of these cultures with kinetin, a plant hormone has resulted in relatively strong corrective effects on the FD neuronal cells, demonstrating that the iPSC-based drug discovery approach has good predictive value—although the full clinical proof-of-concept is still far away.

Thus, the patient-specific iPSCs offer a unique opportunity for studying and modeling the effects of specific gene defects on human neuronal development in vitro and for testing small molecules or other potential therapies for the relevant genetic disorders of the nervous system.

## 5. ESC and iPSC-Derived Cardiomyocytes in Drug Discovery

Cardiovascular disease is considered as one of the major and leading cause of death. Since adult cardiomyocytes have a limited regenerative capacity, their loss permanently compromises myocardial contractile function leading to loss of cardiac function and heart failure. Efforts are being made to develop different ways of treating cardiovascular diseases that involves not only perfecting the production of immunocompatible cardiomyocytes but also the establishment of more sophisticated cellular drug discovery and test systems.

Rodent models fail to mimic the basic physiological functions of heart due to their having a much faster heart beat than human; thus they make unreliable animal models to test the drugs for arrhythmias in human. Generation of iPSCs from patients suffering congenital heart disease and their differentiation into cardiomyocytes has been predicted to serve as a model system to study disease pathogenesis and for drug discovery [55]. ESC- and iPSC-derived functional cardiomycytes can potentially improve the efficiency of the drug discovery and screening process [56–58]. Despite the immature sarcomeric and myofibrillar organization, the ultrastructural and morphological similarities between the in vitro derived cardiomyocytes and adult heart cardiomyocytes make them the better choice model for drug discovery [58–61]. Given that pluripotent cells can be differentiated into cardiac cells that form a functional syncytium in vitro within which action potential propagation is synchronized, previously unavailable models can be engineered for studying the effect of potential cardioactive drugs in the electric conduction in human cardiac tissue [62]. To build such models, which might substitute for whole animal experiments, differentiated cells have to be grown on the surface of multielectrode arrays (MEAs) [63]. The advantages of using in vitro differentiated cardiomycytes include their ability to keep the contractile function thereby providing the homogenous cell culture for screening which ultimately contributes to improved high-throughput drug discovery process. ESCs and iPSCs differentiated in vitro into cardiomyocytes are being used by pharmaceutical companies to screen compounds involved in several biological processes.

## 6. ESCs and iPSC-Derived Neurons in Toxicity Testing

Toxic effects of chemical compounds, environmental changes, and naturally occurring substances can lead to neurotoxicity which, in turn, leads to temporary or permanent harm to the central or peripheral nervous system. In case of excitotoxicity, a specific form of neurotoxicity, excessive stimulation of the neurons occurs due to spinal cord injury, stroke, or traumatic brain injury during which neurotransmitters like glutamate and similar substances are responsible for the damage and death of nerve cells. Environmental toxicants or pharmaceutical agents can influence such excitotoxic processes and can exaggerate their deleterious effect. Therefore, it is of utmost importance to develop more predictive cell-based models and powerful screening tools for assessing the neurotoxicity of chemical compounds, drug candidates, and environmental agents. Human neurons derived from ESCs and iPSCs can be attractive models to study the neurotoxicity. The ESC- and iPSC-derived neurons exhibit functionality and behavior of mature neurons and are available in large quantities. It is possible to develop live cell assays that allow characterization of neurons and neuronal networks for health and extent of the connectivity. The neurotoxicity test models will allow for studying on one hand the adverse effect of drug candidates on neuronal cells and on the other hand the general neurotoxicity in assays that are well suited for screening of lead compounds and potentially important for reducing animal experimentation and the cost of preclinical development.

One variable that can be used in neurotoxicity tests and can be easily turned into an assay endpoint is the amount and changes of intracellular calcium. Several toxicants have already been shown to have an effect on intracellular calcium homeostasis, which is a reliable indicator of neuronal health and undisturbed function. Developing a system to visualize intracellular calcium levels may shed light on similar toxic effects of previously uncharacterized substances. Currently, cell lines such as PC12 are typically used for the analysis of calcium signaling with the purpose of determining the complex cellular changes triggered by environmental and pharmacologic neurotoxicants [64]. However, scientific consensus exists that this immortalized cell line does not recapitulate the phenotypic features of neurons. Murine primary neuronal cultures could be more adequate to address such questions, but unfortunately the lifespan of such cultures is typically 2-3 weeks, and their establishment requires sacrificing pregnant mice and dissecting their embryos. Several studies have demonstrated the usefulness of calcium measurements in assessing the toxicity of various compounds. For instance, observing a decrease in the depolarization-elicited calcium elevations that accompanies the release of the specific neurotransmitter synthesized by a given neuron can provide valuable information about the toxicity of an uncharacterized compound. A similar approach has been used to reveal the toxic effects of hexabromocyclododecane (HBCD), a substance that is not biodegradable under aerobic conditions and is very toxic to aquatic organisms [64]. Calcium signals can also uncover excitotoxic effects that previously uncharacterized substances might have. Excitotoxicity is accompanied by an abnormally high level of neuronal activation and a concomitant increase in intracellular calcium, for instance, has been found to underlie the neurotoxic effects tributyltin, a substance used as a heat stabilizer, agricultural pesticide, and component of antifouling paints [65]. The relevance of calcium measurements for environmental toxicity is exquisitely exemplified by a study revealing that neurotoxicants such as manganese, lead, and benzo(a)pyrene (a product of incomplete combustion) alter the properties of intracellular calcium waves [66].

Neurotoxicity screens using test systems (TSs) such as two-dimensional neuronal culture with a defined percentage of neuronal subtypes or three-dimensional culture [67, 68] differentiated from pluripotent cells are under development. The TSs are studied by test methods (TMs) defining a set of variables (such as neurite length for example) to measure the changes occurring in response to the tested substance. Reference compounds are typically selected from a list of recommended compounds for developmental neurotoxicity test validation [69]. Comparing cytotoxic or apoptosis-triggering effects of a given compound at different stages of neuronal differentiation can also provide useful information about specific developmental neurotoxicity [70].

## 7. ESC and iPSC-Derived Cardiomyocytes in Toxicity Testing

Pluripotent stem cells can be spontaneously differentiated into beating cardiomyocyte-like cells. The current cardiac lineage differentiation system from pluripotent stem cells includes use of spontaneous embryoid body (EB) formation in suspension culture, coculture of pluripotent stem cells with mouse endoderm-like cells (END-2 cells), and directed differentiation towards cardiac lineage using defined growth factors (such as BMP-4 or activin) either in suspension or in monolayer culture (REFs).

Cardiotoxicity can lead to the formation of reactive oxygen species (ROS), apoptosis, altered contractibility, change in cardiac rhythm, and altered cardiac gene expression, which can be life threatening or may lead to long-term alterations of cardiovascular functions. Of the 40% of drug failures during the clinical trials [71, 72], 19% drug withdrawal has been observed due to cardiotoxicities—thus it is extremely important to test the safety and efficiency of the drugs using an appropriate model system [73]. Development of high-throughput technologies (where more than, 100 molecules can be tested) for drug screening, evaluation and toxicity testing can help to identify the best compound, and improve the efficiency of drug discovery, and save economic loss [71].

In many cardiotoxicity cases, a direct interaction of drugs with specific ion channels expressed by the cardiomyocytes leads to alteration in ion conduction through these specific channels. Drug effects on potassium currents could lead to QT-prolongation, potentially fatal arrhythmias and sometimes cardiomuscular damage without affecting ion channels [42, 74]. Cancer chemotherapies might cause cardiomyocyte apoptosis and dysfunction; however, different chemotherapeutics might have different toxicity mechanisms [75, 76]. In pharmaceutical industries, the cardiotoxicity test models are based on cell lines, animal cardiomyocytes, and small/large animal models [40, 41]. Failure of animal tissue model systems to respond to the drugs in a similar manner as human tissue responds has motivated some of the pharmaceutical companies to use human ESCs and iPSCs derived cardiomyocytes for the cardiotoxicity testing [40–42]; it is expected that with time other companies will follow this example.

Several protocols and strategies have been reported for in vitro differentiation of cardiomyocytes from ESCs and iPSCs. These cardiomyocytes are functional in vitro and have responded to the drugs in similar way as fetal cardiomyocytes [77]. Usually, a subset of drugs with known and accepted arrhythmogenic properties can be used to treat the ESC- and iPSC-derived cardiomyocytes for functional characterization. Encouraging results have been obtained with the use of electrophysiology for studying the response of the ESC- and iPSC-derived cardiomyocytes to drug treatment. However, these results have limitations due to the different experimental setup and number of drugs in each study.

Human pluripotent stem-cell-derived cardiomyocytes have been used to study drug-induced QT interval prolongation [41, 78]. The drugs including quinidine D, L-sotalol, cisapride, and terfenadine used in this study were associated with QT prolongation and/or torsade de pointes in humans. Furthermore, drugs such as ketoconazole and verapamil were included as negative controls to demonstrate specificity [41]. A detailed dose-response analysis in which expected effects on QT interval overlapped with prolonged field potential duration demonstrated a good starting point, but to obtain a higher level of confidence in this system and to gain industry acceptance, a larger collection of drugs needs to be evaluated, and the cells have to be produced under standard operating procedures (SOPs) governed by an industry standard quality assurance and quality control system. Alternatively, for the comparison of the drug effects, two recent studies [79, 80] have demonstrated concordance between hiPSC-derived cardiomyocytes and conventional, well-validated, rabbit and canine ex vivo Purkinje fiber models, which are commonly used as follow-up assays. The transmembrane action potential of the ESC- and iPSC-derived cardiomyocytes has been studied using microelectrodes [80]. The drug-induced arrhythmic events were assessed by using reverse use dependence, triangulation of the action potential, and short-term variability of repolarization parameters. The results suggest that the rabbit Purkinje fibers and ventricular-like ESC- and iPSC-derived cardiomyocytes responded in a similar way with regard to the incidence of early after-depolarization, increased triangulation, and short-term variability of repolarization in response to the human ether-a-go-go–related gene (hERG) channel blocking compound E-4031.

Another study demonstrated that ESC- and iPSC-derived cardiomyocytes provided exceptional pharmacological sensitivity in an action potential assay when compared with rabbit and canine Purkinje fibers [79]. Additionally, these cardiomyocytes resulted in reduced compound consumption, cost and time savings compared to the conventional Purkinje fiber assays. The study also reports that these cardiomyocytes are good detectors of proarrhythmic events and that soon after depolarization were induced by the reference compounds terfenadine, sotalol, cisapride, and E-4031. It is known that the compounds that do not interfere with ion channel functionality can also cause cardiotoxic insults. The ESC- and iPSC-derived cardiomyocytes are considered to be well suited to study the effects of compounds which do not interfere with the ion channel functions but still cause cardiotoxicity, an effect that cannot be revealed by using the conventional cell line and receptor overexpression-based approaches [41].

The ESC- and iPSC-derived cardiomyocytes have recently been used to study doxorubicin-triggered toxicity [81]. Two clinically decisive biomarkers of cardiac damage that are sensitive indicators for doxorubicin-induced toxicity were studied. The ESC- and iPSC-derived cardiomyocytes released detectable levels of cardiac troponin T and fatty acid-binding protein 3 in a dose-dependent manner after doxorubicin induction [82]. Based on the availability of very sensitive and rapid analytical tools for these biomarkers, the assay lends itself well to miniaturization and high-throughput formats. This strategy demonstrates that molecular mechanisms of cardiotoxicity after drug treatment can be studied.

## 8. Future Challenges and Perspectives

One of the important tasks in using ESCs and iPSCs for drug discovery and toxicity testing is producing large amount of cells with low heterogeneity that behave in a consistent way. The robotic and suspension culture methods for ESCs [83, 84] provide an extensively evaluated and standardized system for ESC culture. Although derivation of iPSCs is considered as interesting and superior model system for the development of drug discovery and toxicity testing model system, several obstacles related to the reprogramming procedure have to be overcome. To increase the yield of reprogrammed cells, the choice of cells to be reprogrammed is of particular importance, given the variable reprogrammability of the different cell types [24, 85–87] (Tat et al. [20]). Furthermore the choice of reprogramming factors and gene delivery methods will constitute a cornerstone for each and every drug discovery or toxicity testing application.

The next challenge would be to optimize the protocols for the differentiation of ESCs and iPSCs into neuronal and cardiac lineages, though several protocols have been used to produce functional neurons and cardiomyocytes from the ESCs and iPSCs, albeit with varying efficiency. Understanding the different signaling pathways and factors responsible for cardiac and neuronal differentiation of these cells would help to improve the differentiation protocols. Development of more homogenous cell phenotypes after differentiation is a necessity for the development of reliable and well-standardized model system for drug discovery and toxicity testing. Moreover, production of more mature cell types from pluripotent stem cells can be beneficial for use as a model system for adult human organs in pharmacological and toxicological studies. Culture of the differentiated neurons and cardiomyocytes may help to produce more appropriate cell types for testing pharmacological compounds.

In conclusion, the use of ESC- and iPSC-derived neurons and cardiomyocytes for drug discovery and toxicity testing constitute an extremely promising tool when more sophisticated, efficient, and reproducible methods of differentiation and for scaling up are be achieved. The early “proof-of-concept” examples presented in this paper highlights the trend and the potential of the approach; however the current industrial and medical practice is still based on the properly validated “old” technologies. Further major efforts and commitment will be necessary to fulfill the expectations towards pluripotent stem cell-based systems—international efforts including the recent European Union 7th Framework Programme “Innovative Medicine Initiative” calls for human iPSC-based drug and toxicity testing systems hold good promise to finance and stimulate such developments.
